# Metabolic profiling reveals local and systemic responses of kiwifruit to *Pseudomonas syringae* pv. *actinidiae*

**DOI:** 10.1002/pld3.297

**Published:** 2020-12-16

**Authors:** Yawei Li, Xiaojie Wang, Yunliu Zeng, Pu Liu

**Affiliations:** ^1^ Anhui Engineering Laboratory for Horticultural Crop Breeding College of Horticulture Anhui Agricultural University Hefei China; ^2^ Key Laboratory of Horticultural Plant Biology of Ministry of Education College of Horticulture and Forestry Sciences Huazhong Agricultural University Wuhan China

**Keywords:** *Actinidia*, bacterial canker, metabolic profile, *Pseudomonas syringae* pv. *actinidiae*

## Abstract

*Pseudomonas syringae* pv. *actinidiae* (*Psa*), a bacterial pathogen, causes bacterial canker disease in kiwifruit. To elucidate the local and systemic influences of *Psa* infection on kiwifruit, comprehensive analyses were conducted by combining metabolomic and physiological approach under *Psa*‐infected treatment and mock‐inoculated control in leaves, stems, and bleeding saps. Our results show that *Psa* infection stimulated kiwifruit metabolic reprogramming. Levels of many sugars, fumarate, and malic acid were decreased in *Psa*‐infected leaves and stems, accompanied by the increased level of amino acids (AAs), which implies the anaplerotic reaction to replenish the TCA cycle generating energy and intermediates for defense‐related metabolic pathways, such as phenylpropanoid metabolism. The inconsistent results were observed in bleeding saps, which may be attributed to the induced phloem transport of carbon (C) out of leaves and such a transport benefits bacterium movement. Arg, Gln, and pyroglutamic acid systematically were accumulated in long‐distance leaves, which probably confers to systemic acquired resistance (SAR) and *Psa* inoculation accelerated the nitrogen (N) cycling in kiwifruit. Moreover, *Psa* infection specifically affected the content of phenolic compounds and lignin. Phenolic compounds were negatively and lignin was positively related to kiwifruit *Psa* resistance, respectively. Our results first reveal that *Psa* enhances infection by manipulating C/N metabolism and sweet immunity, and that host lignin synthesis is a major physical barrier for restricting bacterial infection. This study provides an insight into the complex remodeling of plant metabolic response to *Psa* stress.

## 1 INTRODUCTION

Kiwifruit, the genus of *Actinidia*, is an economically important fruit crop with primary production in China, Italy, New Zealand, and Chile. As an economically damaging trunk bacterial disease, bacterial canker of kiwifruit caused by the bacterial pathogen *Pseudomonas syringae* pv. *actinidiae* (*Psa*) is a major constraint to the production of kiwifruit. The cultivars of *A. chinensis* var. *chinensis* and *deliciosa* are generally sensitive to *Psa*, such as “Hort 16A,” “Jintao,” and “Hongyang.” As an epiphytic bacterium, the pathogen of *Psa* can be colonized on the surface of the plant tissues, including flowers/pollen, leaves, and stems, but it cannot induce any visible symptoms until an “optimal state” is achieved (Donati et al., 2018; Straub et al., 2018). After it enters the kiwifruit by natural orifice (stomata) or wounds, *Psa*, as an endophytic phytobacterium, can infect the flowers/pollen, leaves, and stems, then successively colonizes and occludes the vascular system (xylem vessels and phloem sieve tubes) for systemic infection (Vanneste, 2017; Xin et al., 2018), causing symptoms including dark brown spots surrounded by yellow halos on leaves, shoots dieback, wilting of buds, and oozing of opalescent or red rusty exudates on canes and trunks (Everett et al., 2011; Yuichi et al., 1989). The infected male flowers produced contaminated pollen, which could transmit *Psa* to healthy plants. Meanwhile, pollinators (*Apis mellifera* and *Bombus terrestris*) were reported to be contaminated with *Psa* (Donati et al., 2018). Previous studies reported that when average temperatures fluctuated between 10 and 18°C for more than 10 days, *Psa* infections progressed rapidly, that *Psa* had an optimal growing temperature of 15°C (±3°C). The infection and production of exudate decreased progressively as the temperature rose from 18 to 23°C, and that above 25°C, the infection did not occur (Vanneste, 2017). Therefore, spring and autumn are the most favorable seasons for *Psa* infection (Gao et al., 2016).

The growth of plants, as sessile organisms, is often influenced by abiotic and biotic stresses. Plants have evolved a set of intricate constitutive barriers including biochemical and physiological change, and production of antimicrobial compounds to prevent and defend against pathogen attack. When these barriers fail to prevent entry of pathogen, plants are able to develop an induced resistance, such as systemic acquired resistance (SAR; Sticher et al., 1997). These defenses included pathogen‐associated molecular patterns (PAMPs)‐triggered immunity (PTI), and specific bacterial effector‐triggered immunity (ETI; Návarová et al., 2012). ETI is usually associated with the hypersensitivity reaction (HR) that follows a massive burst of reactive oxygen species (ROS) at pathogen inoculation sites. Many natural metabolites such as the phenolic salicylic acid (SA) act as regulatory components of plant innate immunity processes and SAR. SA is a key regulator of plant basal immunity to biotrophic and hemibiotrophic pathogens. Upon pathogen attack, chorismate‐derived SA accumulates in free and glycosides forms systemically in the foliage and induces the expression of a set of SAR‐related genes (Hartmann et al., 2017, 2018; Wang et al., 2018). Recently, there is an increasing interest in the roles of carbohydrates and amino acids (AAs) in plant immunity. Plant AAs play a central role in plant–bacterial interactions as major growth‐limiting nutrients and as precursors for the production of many plant defense compounds (Zeier, 2013). Carbohydrates produced by photosynthesis are well known for their essential role as vital sources of energy required for defenses and carbon skeletons of organic compounds and storage components, and carbohydrates serve as signals for the regulation of defense genes (Bolouri‐Moghaddam & Van den Ende, 2012; Yamada et al., 2016). In addition, sugars, especially disaccharides sucrose, trehalose, raffinose family oligosaccharides, and fructan play a role in ROS production (Trouvelot et al., 2014).

As the gram‐negative hemibiotrophic bacterial pathogen, *P. syringae* has been studied as a model for understanding host‐microorganism interactions since the early 1980s (Xin & He, 2013). Metabolomics has become a powerful tool for better understanding the biochemical and molecular mechanisms of resistance, providing a comprehensive quantitative and qualitative metabolic picture of a living organism under biotic stress. Nuclear magnetic resonance (NMR), gas chromatography‐mass spectrometer (GC‐MS), and liquid chromatography–mass spectrometry (LC‐MS) are the most common methods for metabolic profiling analyses (Luo, 2015; Moros et al., 2017). Extensive studies have been carried out on the change of metabolites in plant–*P. syringae* interaction (López‐Gresa et al., 2011; Qian et al., 2015). *P. syringae* infection induced the accumulation of hydroxycinnamic acid amides, chlorogenic acid, and rutin in tomato leaves (López‐Gresa et al., 2011). NMR‐based metabolomics showed that the presence of the bacterium *Candidatus* Liberibacter asiaticus has a substantial effect on the metabolite composition of the citrus fruit (Chin et al., 2014). LC‐MS and GC‐MS analysis showed that biosynthetic pathways of acetophenone, xanthophylls, fatty acids, alkaloids, glutathione, carbohydrate, and lipid were affected by *Xanthomonas oryzae* pv. *oryzae* infection in rice (Sana et al., 2010). Differentially accumulated metabolites, including sugars (monosaccharides and oligosaccharides), organic acids (oxalic acid and cumic acid), AA derivatives, and other secondary metabolites (mannitol, octanal, hypoxanthine, daidzein etc.) may participate in the metabolic‐level defense response of soybean to *Phytophthora sojae* infection (Zhu et al., 2018).

In this study, combining ^1^H‐NMR spectroscopy with GC‐MS, we carried out a comprehensive analysis of leaves, stems, and bleeding saps in both infected group and mock‐inoculation control group of *A. chinensis* var. *chinensis* cultivar “Hongyang,” which is very susceptible cultivars to *Psa*. The roles of phenolic compounds and lignin in *Psa–*kiwifruit interaction were further analyzed.

## 2 RESULTS

### 2.1 NMR spectroscopy of kiwifruit extracts response to *Psa* inoculation

To gain insight into the effect of bacterial pathogen on the metabolic homeostasis, comparative metabolomic analysis using NMR was performed to quantify the primary metabolites from independent sets of *Psa*‐infected and mock‐inoculated plants of the susceptible cultivar “Hongyang.” From the 42 spectra, a total of 41 metabolites were detected and quantified (Figure S1), including amino acids (AAs), amines and ammonium compounds, organic acids, sugars, and other metabolic intermediates (epicatechin, myo‐inositol, 1,3‐dimethylurate, methanol, UDP‐galactose, and UDP‐glucose). Quantification of the metabolites was performed by comparing the signal integral with the reference integral, and quantities were expressed as mg/g. FW for leaves and stems samples, and mM for bleeding sap samples. The concentration information of all metabolites was listed in Table S1.

**FIGURE 1:**
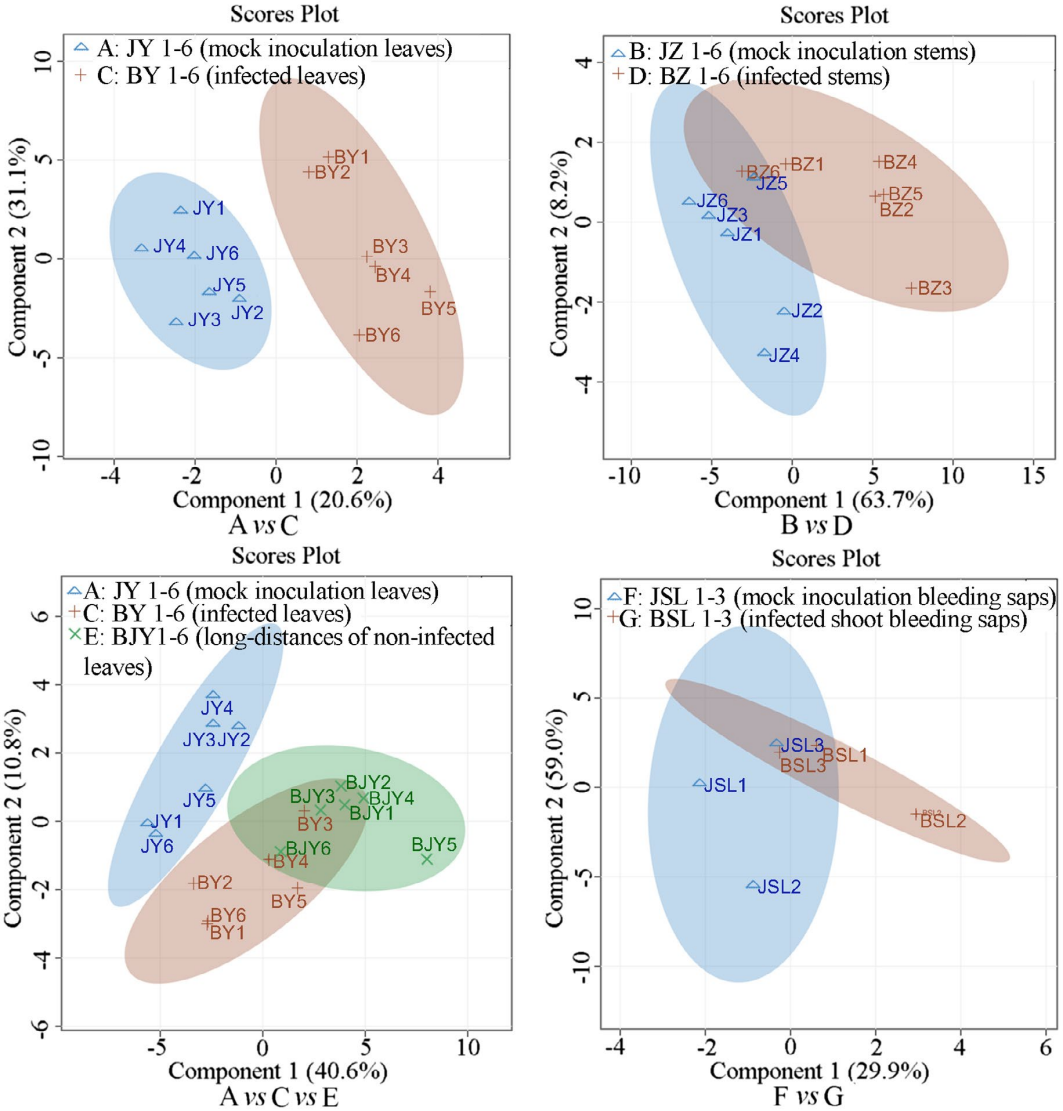
Partial least square‐discriminant analysis score plots of metabolomic changes in *A*. *chinensis* var. *chinensis* “Hongyang” by nuclear magnetic resonance

To analyze the metabolic data of *Psa‐*infected group and mock‐inoculated control group under biotic stress, partial least square‐discriminant analysis (PLS‐DA) were conducted with the NMR spectral sets. Significant differences were observed between mock‐inoculation leaves (A) and infected leaves (C)/leaves with no symptoms at a distance from the inoculation point (E; *R* ^2^ of 0.948 and a *Q* ^2^ of 0.6996); mock‐inoculation stems (B) and infected stems (D; *R* ^2^ of 0.7475 and a *Q* ^2^ of 0.4771), which suggested that *Psa* infection resulted in a clear metabolic shift on leaves and stems. However, partial overlapping between C and E, mock‐inoculation bleeding saps (F), and infected shoot bleeding saps (G) were observed, indicating that there were similar metabolic processes among those groups (Figure 1). Those results explained why there were less significant differences between local and systemic leaves (SAR), infected and non‐infected stem exudates, which was consistent with the results of *Arabidopsis–P. syringa*e interaction (Návarová et al., 2012).

**FIGURE 2:**
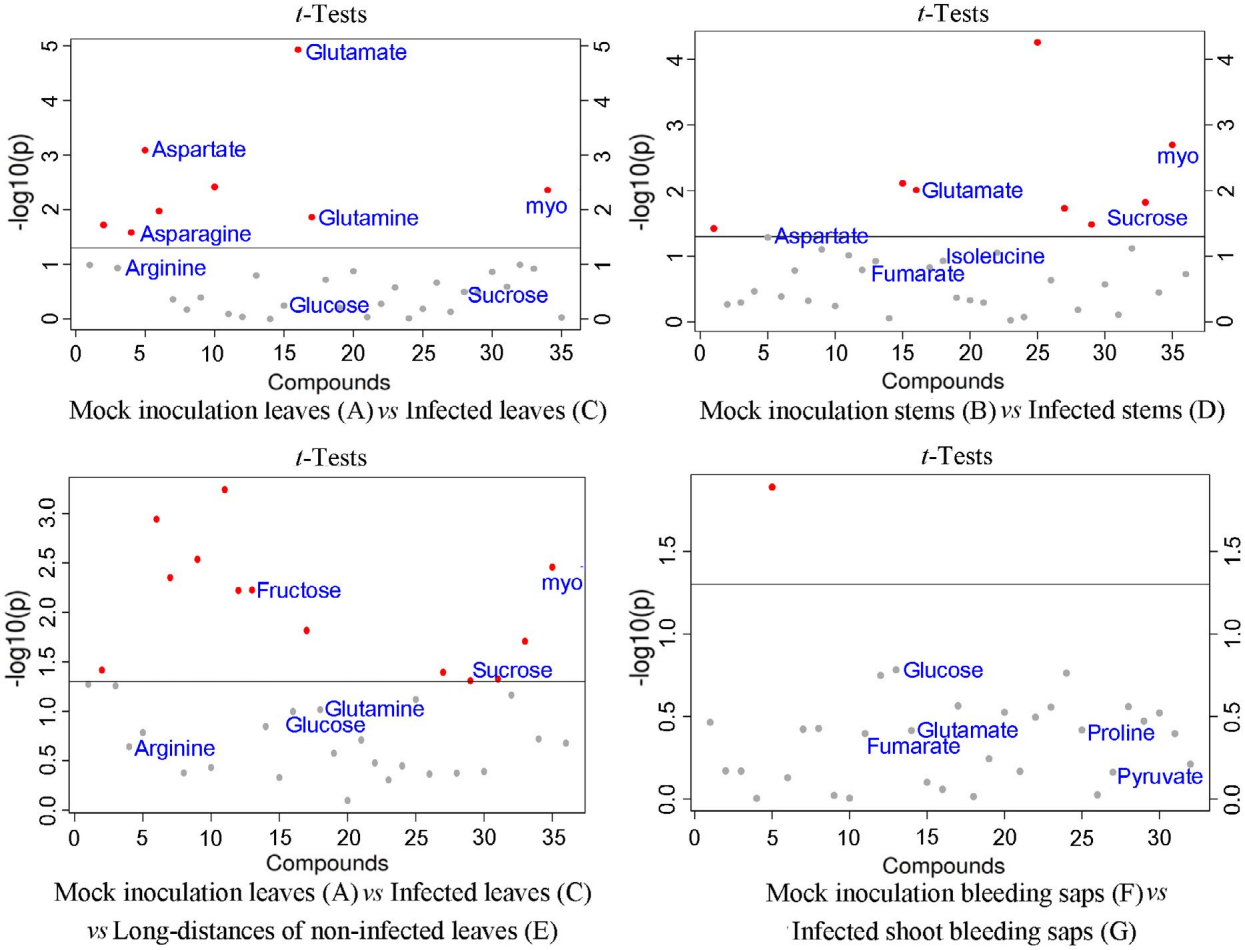
ANOVA score plots of metabolomic changes in *A*. *chinensis* var. *chinensis* “Hongyang” by nuclear magnetic resonance. All the metabolites (VIP > 1.0) were represented by points. The gray points indicate *p* > .05 and the red points indicate *p* < .05 using *t* test (*n* = 6)

The loading plot generated from the PLS‐DA reflected the contributions of the different metabolites from the treatments to the differences (Figure 2). The points farther away from the center contributed more than the nearby plots. The variable importance in projection (VIP) values were calculated through PLS‐DA. The larger the VIP values were, the greater their contributions were. Generally, VIP > 1 represents significant differences. On the basis of the loading plot, when the VIP values > 1.0, and the *p* ˂ .05, the points representing myo‐inositol, sucrose, glucose, arginine (Arg), asparagine (Asn), aspartate (Asp), glutamine (Gln), and glutamate (Glu) were identified in A (mock leaves) versus C (local leaves). Among them, *Psa* infection significantly increased the levels of Gln, Glu, Arg, Asp, and alanine (Ala), but reduced myo‐inositol, sucrose, and glucose in leaves. The points representing sucrose, myo‐inositol, fumarate, Glu, Asp, and isoleucine (Ile) were identified by comparing B (mock stems) and D (local stems). Consistent with the leaf comparison result between A and C, *Psa* infection significantly reduced the levels of myo‐inositol, sucrose, and Glu, and increased the levels of Asp. By comparing F (mock shoot bleeding saps) and G (local shoot bleeding saps), the representative points of glucose, fumarate, Glu, Pro, and pyruvate were identified in bleeding saps (VIP > 1, Figure 2). The content of Glucose, Pro, and pyruvate was higher in group G, but the content of fumarate and Glu was lower.

### 2.2 GC‐MS analysis of kiwifruit extracts response to *Psa* inoculation

**FIGURE 3:**
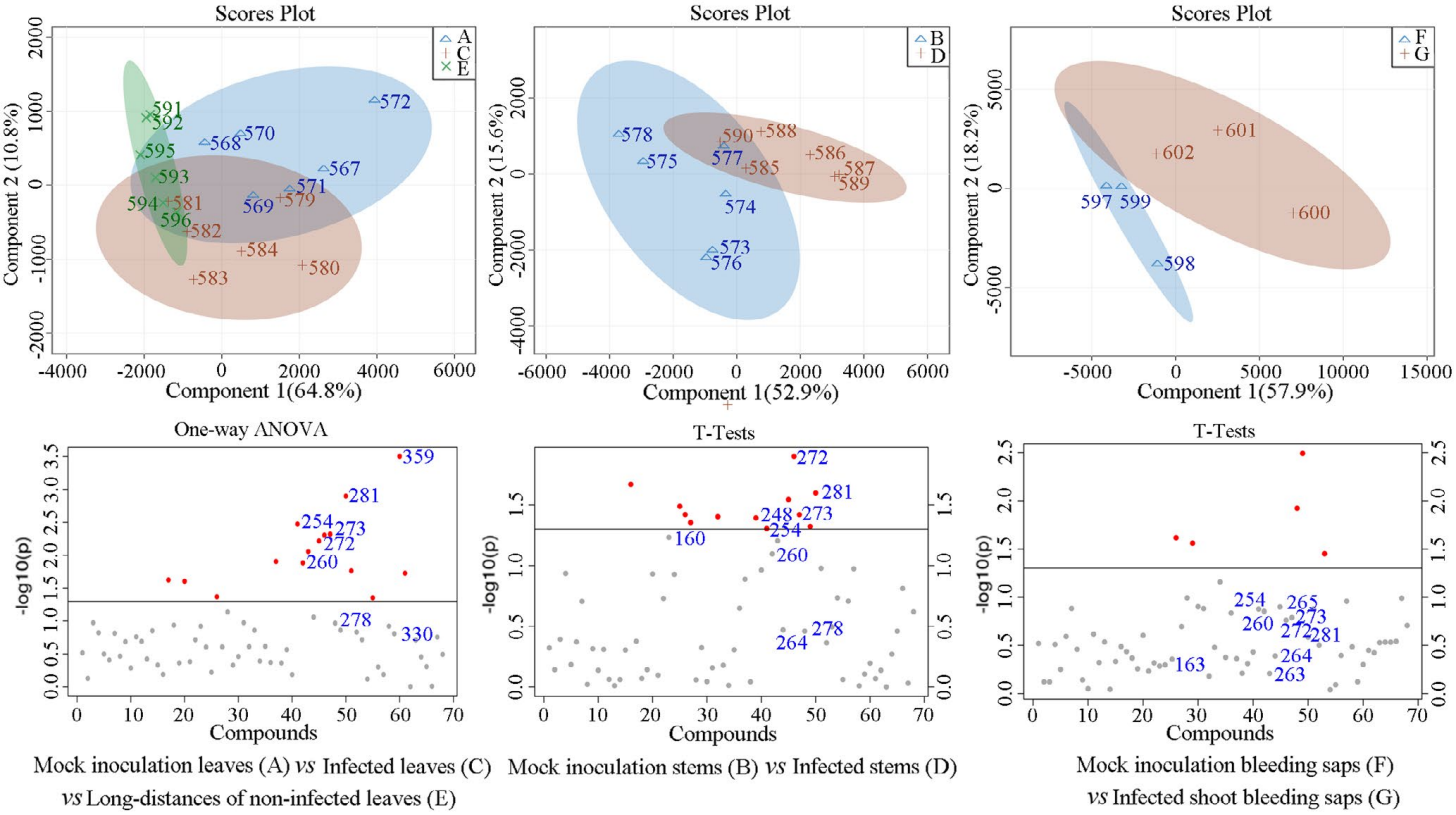
Partial least square‐discriminant analysis score plots and ANOVA of metabolomic changes in *A*. *chinensis* var. *chinensis* “Hongyang” by gas chromatography‐mass spectrometer. The metabolites represented by each number are given in the Name List of Table S2. All the metabolites (VIP > 1.0) were represented by points. The gray points indicate *p* > .05 and the red points indicate *p* < .05 using *t* test (*n* = 6)

Gas chromatography‐mass spectrometer provided a complementary approach to identify small polar metabolites associated with plant responses to *Psa* infection. By GC‐MS, a total of 425 metabolites were detected, and on the basis of retention index and mass spectrum, 68 metabolites were identified by the Binbase identifier (Table S2; Figure S2). Consistent with the NMR spectral sets, the score plot of PLS‐DA indicated that *Psa* infection obviously induced the alteration in production of metabolites in leaves, stems, and bleeding saps, which was different from the tendency exhibited by the control group (mock leaves (A) vs. local leaves (C) vs. systemic leaves (E); mock stems (B) vs. local stems (D); mock shoot bleeding saps (F) vs. local shoot bleeding saps (G)). Those results indicated that some metabolite content levels changed when kiwifruit was infected by *Psa* in leaves, stems, and bleeding saps (Figure 3).

Sorbose (254), galactose (272), psicose (260), idose (273), mannose (278), glucose (281), glycerolaldopyranosid (330), and helicin (salicylaldehyde‐beta‐d‐glucopyranoside; 359) were identified in leaves by comparing A with C/E with the VIP values > 1.0 and the *p* ˂ .05 (Figure S1). Only helicin, the precursor of salicylates, displayed higher content in *Psa*‐infected leaves than that in control group leaves, while the content levels of sorbose, galactose, psicose, idose, mannose, glucose, glycerolaldopyranosid decreased in *Psa*‐infected leaves. Furthermore, *Psa* infection significantly increased the levels of pyroglutamic acid (160) and isocitric acid (229) in systemic leaves of kiwifruit.

The content levels of galactose (272), sorbose (254), idose (273), psicose (260), mannose (278), quinic acid (248), fructose (264), glucose (281), epicatechin (410), and N‐(3‐aminopropyl) morpholine (173) in infected stems were lower than those in mock stems (VIP > 1.0; *p* ˂ .05), while the levels of pyroglutamic acid (160) in infected stems were higher than that in mock stems.

The metabolites of sorbose (254), idose (273), psicose (260), galactose (272), psicose (263), 2‐oxo‐gulonic acid (265), and glucose (281) were increased, whereas fructose (264), pyroglutamic acid (163), and succinic acid (97) were decreased after *Psa* infection (VIP > 1.0; FDR ˂ 0.05) in kiwifruit bleeding saps, compared with those in mock group.

### 2.3 Comprehensive analysis of GC‐MS and NMR data

**FIGURE 4:**
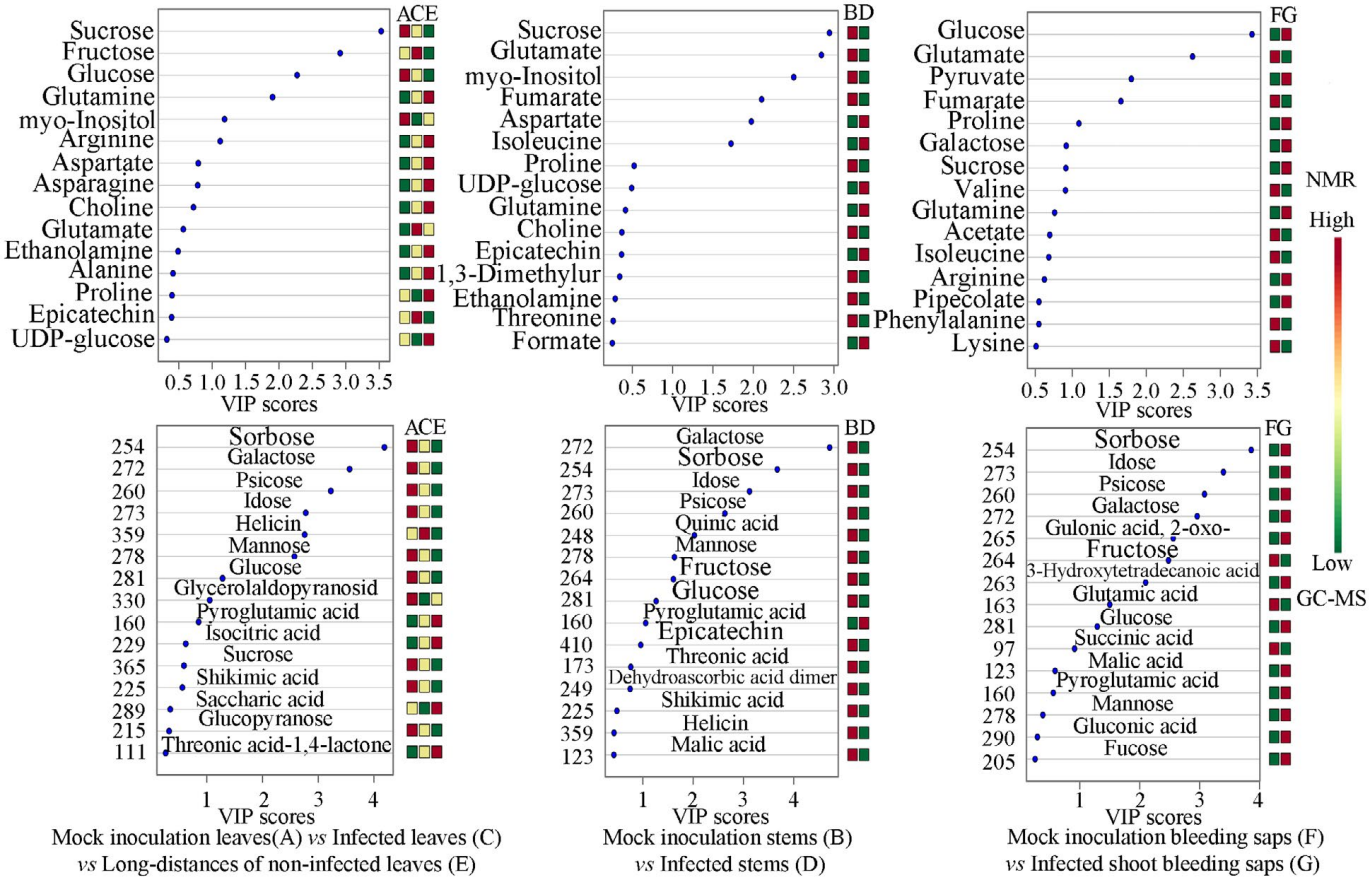
VIP (variable importance in projection) score plots of metabolomic changes in *A*. *chinensis* var. *chinensis* “Hongyang” by nuclear magnetic resonance (NMR) and gas chromatography‐mass spectrometer (GC‐MS; *n* = 6). The color box on the right reflects the concentrations of this metabolite in different groups. Red, yellow, and green indicate the concentration range from high to low, respectively

By combing the data of NMR and GC‐MS, a total of 18, 16, and 13 metabolites were enriched in leaves, stems, and bleeding saps, respectively. Among them, glucose, galactose, sorbose, idose, psicose, fumarate, and Glu altered in all tested tissues. In addition, sucrose, mannose, myo‐inositol, Asp, and Ile were observed in both leaves and stems. Metabo Analyst 4.0 (Chong et al., 2018) analysis indicated that a total of 28, 26, and 21 pathways were identified (Table S3), respectively, in leaves, stems, and bleeding saps after *Psa* infection, including AA metabolism, nitrogen (N) metabolism, starch and sucrose metabolism, tricarboxylic acid (TCA) cycle, carbon fixation in photosynthetic organisms, glutathione metabolism, and porphyrin and chlorophyll metabolism (Table S3). The metabolites ethanolamine, Arg, Asn, Gln, glycerolaldopyranosid, and helicin were induced merely in leaves, whereas quinic acid, epicatechin, and N‐(3‐aminopropyl) morpholine were induced solely in stems (Figure 4).

### 2.4 *Psa* infection induces systemic changes in metabolite composition

*Psa*‐infection significantly induced metabolic difference in systemic leaves of kiwifruit “Hongyang” by comparing A with C/E, and sucrose, glucose, my‐inositol, Gln, Glu, Arg, Asn, Asp, Ala, choline, and ethanolamine were identified (VIP > 1.0; *p* ˂ .05). Among them, *Psa*‐infection significantly increased the contents of Gln, Arg, and pyroglutamic acid in the distant leaves, but reduced the contents of myo‐inositol, sucrose, glucose, galactose, sorbose, idose, psicose, and mannose (Figure 4). The results indicated that *Psa*‐infection directly/indirectly inhibited carbon fixation in photosynthetic organisms, starch and sucrose metabolism, and fructose and mannose metabolism. Particularly, the low concentration of the myo‐inositol was observed in local and distant leaves of *Psa*‐infection plant, which was consistent with the previous study results of its role in cell wall biosynthesis and stress response (Valluru & Van den Ende, 2011).

### 2.5 *Psa* infection induces metabolic changes in xylem

The xylem in plants is the main conduit for water and minerals from roots to shoots. It is composed of cell wall materials facilitating the movement of water through the plant. The bacterium of *Psa* can systemically colonize in root and stem xylem. Different from those in leaves and stems, glucose, galactose, sucrose, sorbose, idose, psicose, Pro, 2‐oxo‐gulonic acid, pyruvate, 3‐hydroxytetradecanoic acid accumulated in stem exudates from infected branches. Particularly, Pro, 2‐oxo‐gulonic acid, glutamic acid, pyruvate, 3‐hydroxytetradecanoic acid, succinic acid, and fumarate were solely induced in bleeding saps. But fructose, Glu, Val, fumarate, succinic acid, and glutamic acid were reduced. The results showed the tissue‐specific metabolite production after *Psa*‐infection. The pathogen induced not only the disorder of sugar and protein but also leads to metabolic disturbance of energy, immune system, AA, and various metabolic systems. It should be noted that high concentration of sugar is conducive to *Psa* proliferation and spread from pathogen‐inoculation sites.

### 2.6 *Psa* infection induces chitinases activities around the roots

**FIGURE 5:**
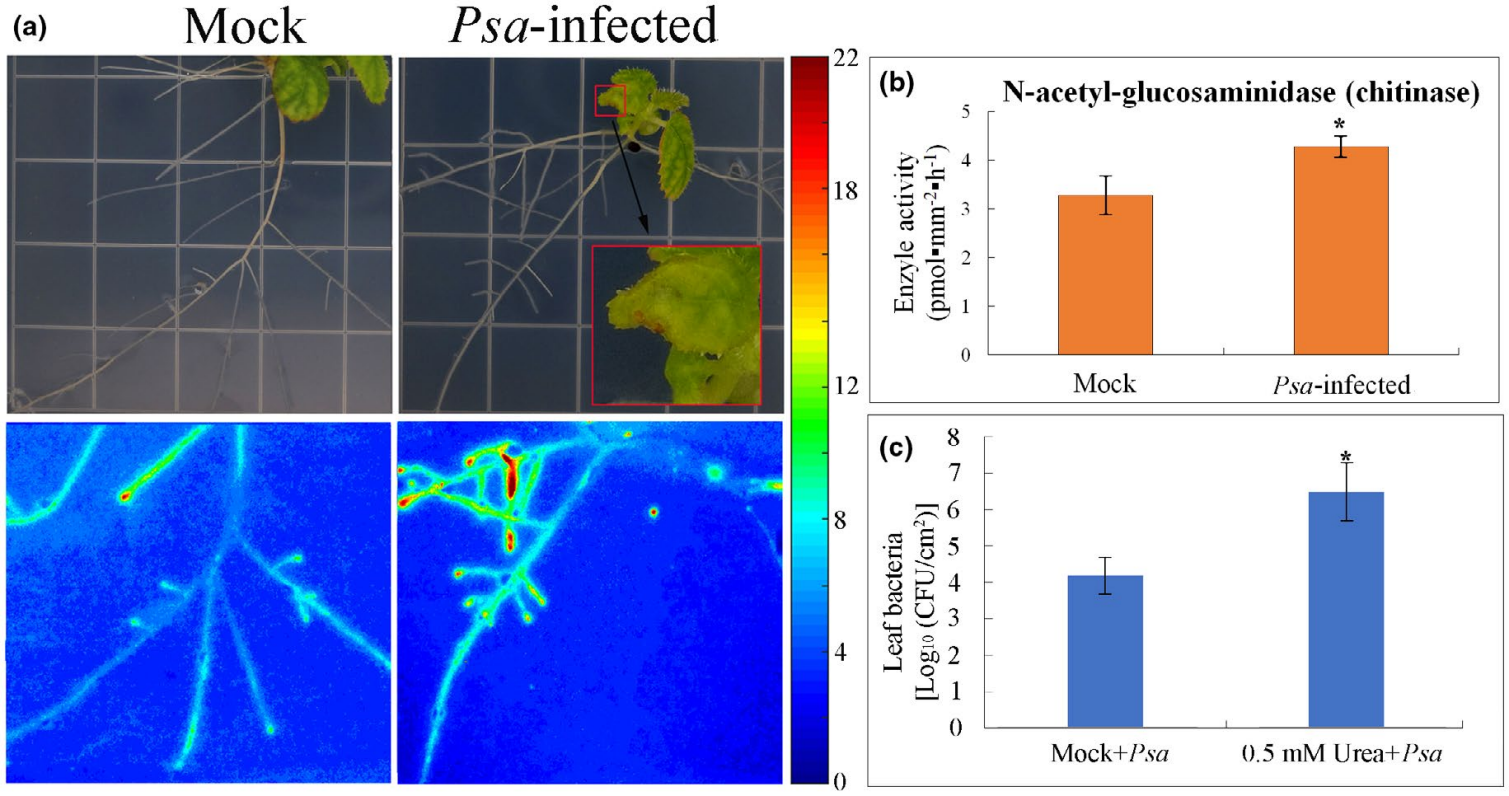
*Pseudomonas syringae* pv. *actinidiae* enhance N cycling of *A. chinensis* var. *chinensis* “Hongyang.” (a, b) *N*‐acetyl‐glucosaminidase (chitinase) activity in living roots 10 days after inoculation of *Psa* in leaf. The calibration line for the chitinase activity is presented on the right. (c) Exogenous N‐supply enhanced plant sensitivity to *Psa*‐infection. Values are the means ± standard (*n* = 3). The asterisks indicate *p* < .05

In situ zymography is a novel method for analyzing extracellular *N*‐acetyl‐glucosaminidases (chitinases) activity in the presence of living kiwifruit root. The spatial and temporal distribution of chitinases activities are involved in plant N cycling in the presence of living roots (Spohn & Kuzyakov, 2014). Plant chitinases are a group of enzymes that hydrolyze chitin that from insects, fungi, or bacteria. Chitinases in plants is a defense mechanism against biological attack (Hadwiger, 2013). The expression of chitinases may be enhanced by bacterial infection (Punja & Zhang, 1993). Chitosan as a product of chitinase could elicit plants lignification and callose deposition (Hadwiger, 2013). Chitinase activity increased and highest at the root tips after *Psa*‐infection 10 days (Figure 5a,b), which indicated that *Psa*‐inoculation accelerated the lignification in kiwifruit root. 0.5 mM Urea ((NH_2_)_2_CO) was added to MSR medium to study the effects of exogenous N‐supply on the resistance of kiwifruit to *Psa* (Figure 5c). The results showed that the susceptibility of kiwifruit to *Psa* increased with the application of urea. This is in accordance with observations in the field, indicating that exogenous N‐supply enhanced plant sensitivity to *Psa*‐infection.

### 2.7 *Psa* infection induces phenolic compounds and lignin changes in kiwifruit

**FIGURE 6:**
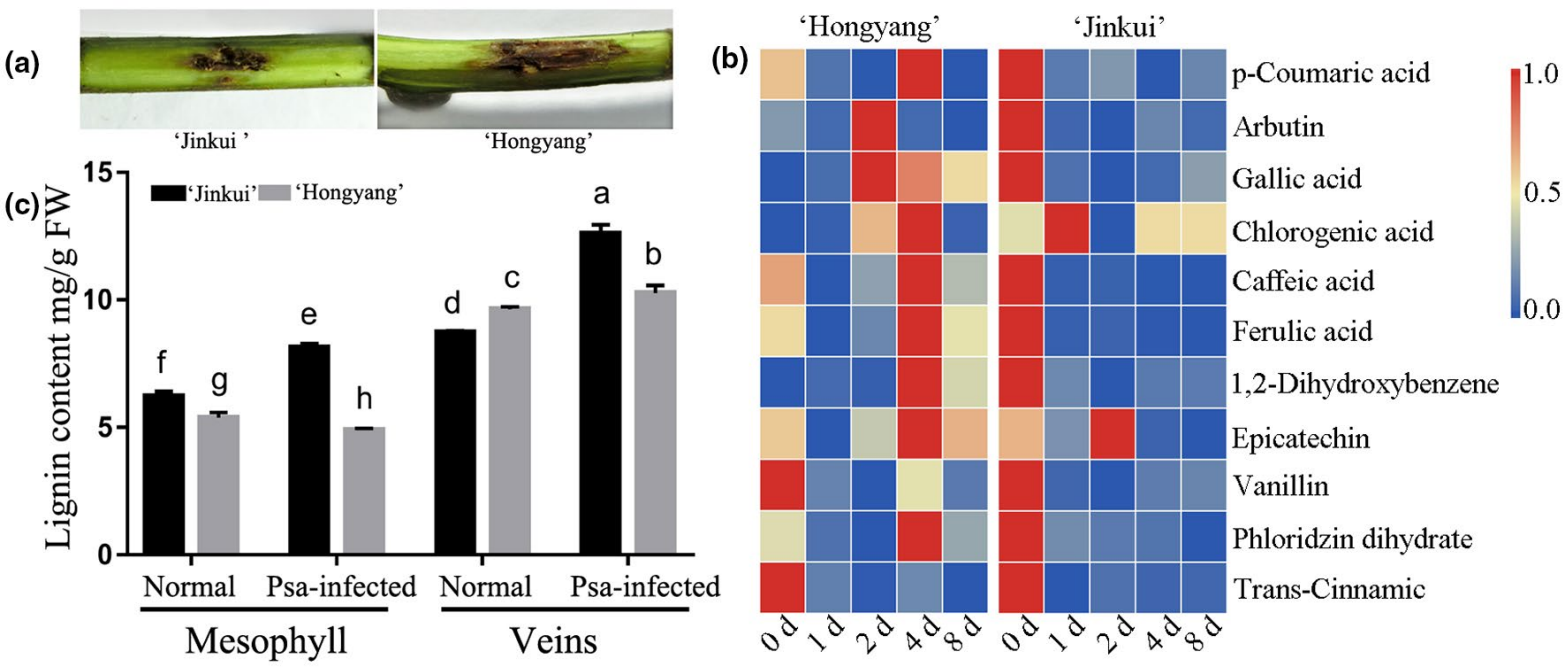
Phenolic compounds and lignin content analysis in “Hongyang” and “Jinkui.” (a) The symptoms of *Psa*‐infected “Hongyang” and “Jinkui.” (b) Phenolic compounds concentration analysis. (c) Lignin content analysis, values are the means + *SE* (*n* = 3). Different letters indicate significant difference at *p*‐value < .05

Plant phenolics, such as flavonoids and lignin precursors, are usually accumulated in response to various biotic and abiotic stresses. Based on the research above, quinic acid and epicatechin have been observed to respond to *Psa* inoculation in leaves and stems (Figure 4). To further reveal their contributions to the disease resistance, the concentrations of individual phenolic compounds were compared between the *Psa*‐resistant cultivar “Jinkui” and the susceptible cultivar “Hongyang” (Figure 6b). A total of 13 phenolic compounds were quantified by HPLC‐DAD. The main phenolic compounds of the resistant cultivar “Jinkui” included 1,2‐dihydroxybenzene, epicatechin, and trans‐cinnamic, whereas epicatechin, trans‐cinnamic acid, *p*‐coumaric acid, and gallic acid represented the major phenolic compounds of the susceptible cultivar “Hongyang.” The genotypes of “Hongyang” presented higher levels of quantified phenolic compounds, compared to those of “Jinkui.” Under *Psa*‐inoculation, most of the phenolic compounds were reduced in “Jinkui.” On the contrary, most phenolic compounds (such as gallic acid, chlorogenic acid, and phenolics) were increased and reached the highest concentration on the 2nd or 4th day in “Hongyang” after *Psa*‐inoculation (Figure 6b). The resistance of genotypes to *Psa* was negatively correlated with the levels of phenolic compounds (Figure 6a,b).

In vitro, the direct activities against *Psa* bacterium of epicatechin, ferulic acid, *p*‐coumaric acid, and caffeic acid were tested. Only the antimicrobial activities of epicatechin at all test concentrations were found. Ferulic acid promoted bacterium growth when its concentration was lower than ≤80 μg/ml while it inhibited the bacterium growth when its concentration was over ≥160 μg/ml. A similar pattern was observed for caffeic acid and *p*‐coumaric acid (Figure S3). Consistent with the above results, phenolics extracted from kiwifruit leaves promoted bacterium *Psa* proliferation with an equal concentration in leaf, which could explain why phenolics might be used directly by *Psa* or they could serve as quorum‐sensing (QS) substances. Phenylalnine ammonialyase (PAL) activity was found to be increased rapidly in “Hongyang,” and was decreased in “Jinkui” at 2 days post‐inoculation (dpi). A similar result was observed for Peroxidase (POD) when “Hongyang” was infected by *Psa*, whereas the activity of POD was obviously lower in “Jinkui” than in “Hongyang” (Figure S4).

As a wood‐infecting pathogen, *Psa* has evolved a system enabling the breakdown of woody tissue of trees. Cell wall is a recalcitrant network of polysaccharides including cellulose, hemicellulose (such as xyloglucan and arabinoxylan), lignin, and pectin (Caffall & Mohnen, 2009). Upon pathogen attack, plant often deposited callose‐rich cell wall appositions (i.e., papillae) at the sites of pathogen penetration, accumulated phenolic compounds and various toxins in the cell wall, and synthesized lignin‐like polymers to reinforce the cell wall (Hematy et al., 2009). In kiwifruit, we found that the content of lignin was obviously induced after *Psa* inoculation in “Jinkui,” but not in “Hongyang” (Figure 6c). The content of lignin in the genotypes of kiwifruit was positively correlated with *Psa* resistance.

## 3 DISCUSSION

At present, bacterial canker of kiwifruit caused by *Psa* has been reported in at least 15 counties, resulting in serious damage (Ye et al., 2019). The *Psa* strains are currently classified into five biovars (biovars 1, 2, 3, 5, and 6), based on the differences in virulence and composition of pathogenicity‐related genes (Vanneste, 2017). Biovar 3 is of strongest virulence among them, and it has posed a major threat to kiwifruit production around the world (Ye et al., 2019). In this study, we performed a comprehensive analysis of local and systemic tissues of kiwifruit to capture a series of metabolite changes associated with disease development following infection with *Psa* by NMR and GC‐MS approaches. A total of 18, 16, and 13 metabolites were identified from leaves, stems, and bleeding saps, respectively, by comparing *Psa‐*infection with mock*‐*inoculated control based on NMR and GC‐MS data (Figure 4). The PLS‐DA result showed that *Psa* triggered significant metabolic changes in local kiwifruit leaves, stems, and bleeding saps, respectively, particularly the change in metabolites such as glucose, galactose, sorbose, idose, psicose (also known as D‐allulose, non‐metabolizable fructose analogue), fumarate, and Glu.

**FIGURE 7:**
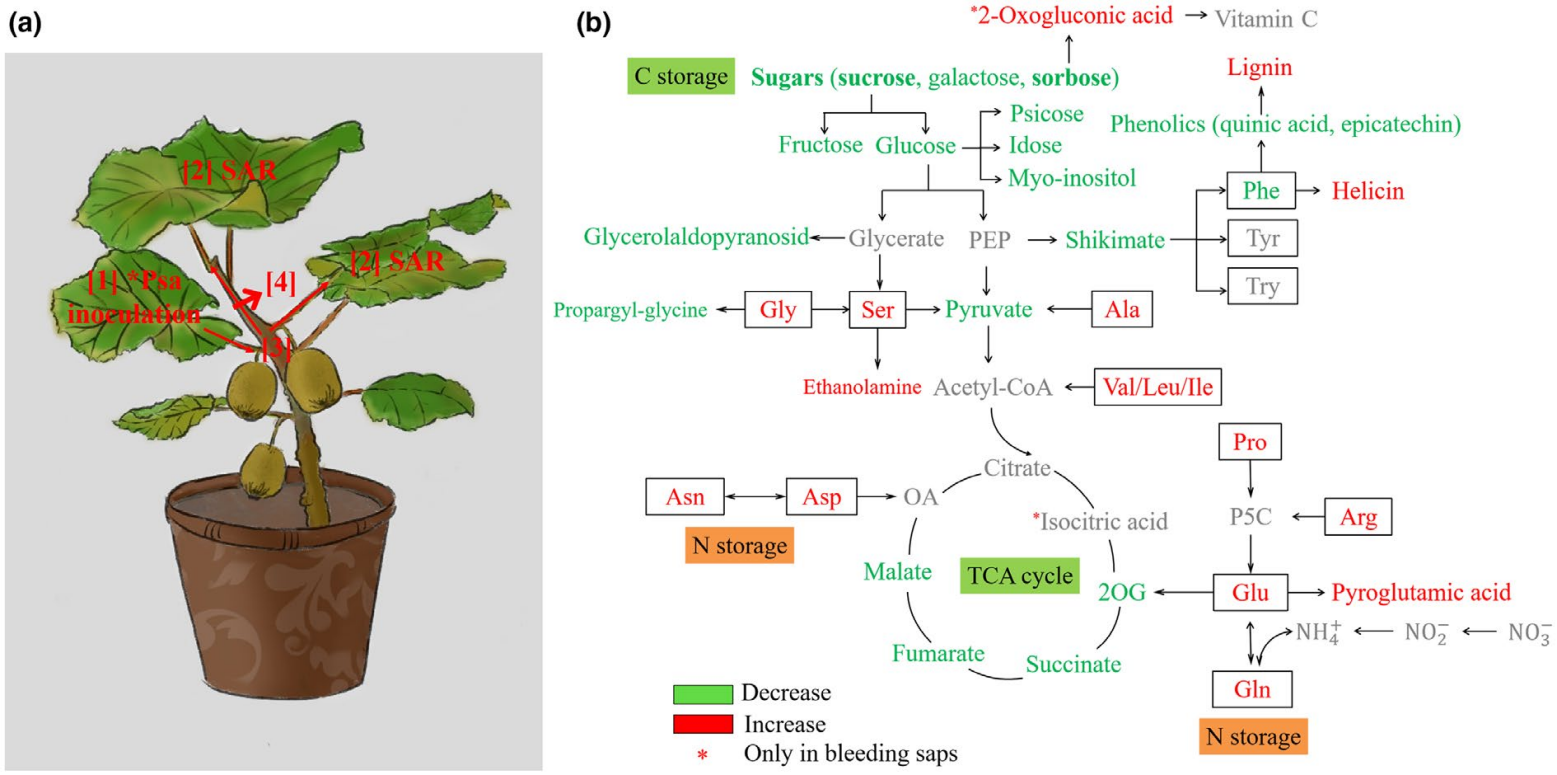
Possible scheme for the metabolism following pathogen attack. The red and blue letters refer to increase and decrease of variables under *Psa*‐infection, respectively; black and gray letters refer to unchanged and unidentified metabolites, respectively. 2OB, 2‐oxobutyrate; 2OG, 2‐oxoglutarate; 3MP, 3‐mercaptopyruvate; ETF, electron‐transfer flavoprotein; ETFQO, electron‐transfer flavoprotein: ubiquinone oxidoreductase; GABA, g‐aminobutyric acid; OA, oxaloacetate; P5C, 1‐pyrroline‐5‐carboxylate; ProDH, proline dehydrogenase; TCA, tricarboxylic acid cycle; PEP, phosphoenolpyruvate

In kiwifruit, *Psa* mainly lived in leaves (apoplasm), shoots, and trunks (vascular system). In leaves, *Psa*‐infection significantly increased the content of Gln, Glu, Arg, Asp, Ala, pyroglutamic acid, and helicin in the leaves, but reduced the content of myo‐inositol, sucrose, glucose, galactose, sorbose, idose, psicose, mannose, and glycerolaldopyranosid. Similar results were observed in stems. Namely, *Psa*‐infection inhibited the accumulation of sugars, fumarate, and malic acid, but increased content of AAs in stems. After *Psa*‐infection, the abovementioned substances in bleeding saps exhibited the change opposite to that in leaves. On the one hand, the results denoted host modulation of primary and intermediary metabolism in response to bacterial infection. On the other hand, plant pathogen that perturbed host central metabolism‐derived nutrients from their hosts to promote its reproduction and virulence via secreting type III secretion effectors (T3SE) or toxins (Rico et al., 2011). The results were consistent with the symptoms of bacterial canker of kiwifruit, including suppression of photosynthesis, and dark brown spots surrounded by yellow halos. Such symptoms led to the reduction in carbohydrates synthesis in the chloroplasts of *Psa*‐infected tissues. In these tissues, carbohydrates supplied the energy and carbon for kiwifruit defenses, since plant defense was highly energy‐demanding processes (Trouvelot et al., 2014), and carbohydrates served as signals for the regulation of defense genes. Plant defense heavily drained TCA cycle‐generated energy and intermediates (Bolton, 2009) to support the costly defense‐related metabolic pathways. For example, phenylpropanoid metabolism consumed up to 20% of the total photosynthetic carbon in plants. This huge demand highlighted the significance of anaplerotic reactions (i.e., filling‐up reactions; Chu et al., 2019), such as metabolism of Glu and Asp. The anaplerotic reactions replenished the cycle energy and ensured the constant functionality of the TCA cycle in these circumstances (Figure 7). Recent transcriptomic data suggest that *Psa* inhibits photosynthesis in “Hongyang” (data not shown). Interestingly, *Psa* infection inhibited the accumulation of most investigated sugars in leaves and stems, but not inhibited in bleeding saps (Figure 7). The inconsistent results might be attributed to the induced phloem transport of carbon out of leaves, which benefited the movement of bacterium form leaves to stems. As precursor of salicylates, helicin (salicylaldehyde β‐D‐glucoside) displayed high content in *Psa*‐infected leaves. Previous studies showed that the hydrolysis of helicon resulted in the release of salicylaldehyde which was toxic to bacteria and served as a defense compound (Caboni et al., 2013).

Bacterial pathogens strategically acquire metabolites from their hosts during infection. The *P. syringae* pv. *tomato* (*Pst*) was reported to promote sugar efflux to the apoplast by manipulating host plant sugar transporter. Meanwhile, the intervention of host could prevent such metabolite loss via sugars transporters and glycoside hydrolases manipulated by pathogens (Yamada et al., 2016). The key roles of some sugars and the sugar metabolites including sucrose, D‐psicose, D‐allose and the sugar‐like 2,5‐dideoxy‐2,5‐imino‐D‐mannitol (DMDP), a fructose analogue, have been confirmed in stimulating the plant immunity system, such as sweet immunity and sugar‐enhanced defense, and in regulating defense‐related genes (Bolouri‐Moghaddam & Van den Ende, 2012). Sucrose and myo‐inositol were often observed under (a)biotic stresses (Valluru & Van den Ende, 2011). As a major form of carbohydrate from source to sink tissues by the phloem, sucrose emerged as a candidate signaling molecule in plant innate immunity, and sucrose induced isoflavonoids synthesis and enhanced plant resistance against *Fusarium oxysporum* (Morkunas et al., 2005). Sucrose and glucose induced the PR‐gene transcripts, PR‐2 and PR‐5, in *Arabidopsis* via SA‐dependent pathway (Thibaud et al., 2004), and those sugars could be efficiently utilized by *X. campestris* pv. *campestris* via SWEET systems (Zhang et al., 2019). D‐psicose conferred a significant resistance to rice bacterial blight (Kano et al., 2011). D‐idose was reported to have an inhibitory effect on *Caenorhabditis elegans* growth (Sakoguchi et al., 2016). In addition, the response of carbohydrates accumulation to *Psa* infection included physical disturbance of the cell wall.

Amino acids were accurately controlled to be in a steady state in normal plant tissues. Emerging evidence indicated that AAs play crucial roles in plant–bacterium interaction, in growth‐limiting nutrient (N/C metabolism) and phytohormone precursor synthesis (SA and pipecolic acid), and in defense‐related compound synthesis (glucosinolates, N^δ^‐acetyl‐ornithine, and camalexin; Zeier, 2013). The increase in AAs (Gly, Arg, Asn, Asp, Glu, Gln, and Ile) and carbohydrate starvation in leaves and stems reflected the inhibition of N assimilation. Among these AAs, Asn, Gln, and Arg were common compounds for N storage and transport. Our results were consistent with previous study results that *P. syringae* inoculation increased the accumulation of branched‐chain AAs (Val, Leu, and Ile), aromatic AAs (Phe, Tyr, and Trp), and Lysin SAR, whereas decreased Asp in leaves of *Arabidopsis, that P. syringae* pv. *tomato* was well adapted to metabolizing six most abundant AAs into N and C sources, but not those the low‐abundant AAs in tomato (Rico & Preston, 2008). Glu played a pivotal role in plant defense against pathogens (Seifi et al., 2013), and the knockout of LYSINE HISTIDINE TRANSPORTER1 (LHT1) increased the resistance to *P. syringae* (Liu et al., 2010). The downregulation of arginine biosynthesis is associated with the inhibitory effect of phaseolotoxin during the *P. syringae* pv. *phaseolicola*—in vetch interaction. Moreover, our study showed that *Psa* infection increased the content of pyroglutamic acid in leaves and stems. Pyroglutamic acid, a cyclization product of Glu, was a precursor of gamma‐amino butyric acid (GABA). It has been reported that pyroglutamic acid played a role in non‐enzymatic browning reactions by promoting the color formation of carbohydrate, which explained the rusty red rusty exudates on kiwifruit trunks. Our study implied that reducing N concentrations in kiwifruit could decrease susceptibility against *Psa*, and that N redundancy inhibited triacylglycerol (TAG) synthesis and TCA cycle.

The 2‐oxo‐gulonic acid and Pro accumulations were obviously observed in kiwifruit bleeding saps after the inoculation with *Psa*, but not in leaves and stems. One previous studies reported that 2‐oxo‐gulonic acid acting as nitrogen starvation signal triggered a cascade response in cyanobacteria *Anabaena* sp. PCC7120 (Liu et al., 2013). Pro synthesized in the cytosol from Glu was usually induced by abiotic stress in *Arabidopsis* (Seifi et al., 2013). Since the elevations of leaf Pro levels partly depend on an intact SA signaling pathway and ROS, the stress‐induced and exogenous Pro treatment induced ROS formation, SA accumulation, PR gene expression, and hypersensitive cell death.

Plant phenolics, such as flavonoids and lignin precursors, could accumulate in response to various biotic and abiotic stresses. Flavonoids, as precursors, also provided defense against phytopathogens to promote the synthesis of tissue lignification around infection sites. *p*‐Coumaric acid inhibited bacterial growth by repressing the expression of type III secretion system genes (Li et al., 2009; Vargas et al., 2013). Meanwhile, pathogens have evolved mechanisms to degrade, detoxify, and tolerate phenolic compounds in the process of co‐evolution, or pathogens responded to phenolic compounds as signals. Degradation of flavonols (such as quercetin) by bacteria has been well documented (Braune et al., 2001). Arbutin might be involved in fire blight *Erwinia amylovora* resistance in apple (Petkou et al., 2002). Our study showed that *Psa*‐infection specifically affected the contents of phenolic compounds and lignin that phenolic compounds (quinic acid and epicatechin) were negatively related to kiwifruit *Psa*‐resistance, and that lignin was positively related to kiwifruit *Psa*‐resistance. In moderate concentration, ferulic acid, caffeic acid, *p*‐coumaric acid, or crude extract of phenolics from kiwifruit induced bacterium proliferation. Consistent results were observed by enzyme activity analysis.

Systemic acquired resistance is an inducible immune response that confers a broad spectrum of disease resistance against biotrophic and hemibiotrophic pathogens at the whole plant levels (Sticher et al., 1997). SAR is activated by a local primary leaf inoculation with virulent or virulent pathogens, and SAR renders non‐inoculated, distal leaves more resistant to subsequent infection (Mishina & Zeier, 2007). Evidence from several lines indicated that long‐distance communication of SAR was brought about by metabolic signals that were transmitted from inoculated leaves to distant tissue via the shoot vasculature. Several compounds have been reported to participate in SAR long‐distance communication. These compounds included the putative lipid transfer protein DEFECTIVE IN INDUCED RESISTANCE1 (DIR1), the methyl ester of SA (MeSA), glycerol‐3‐phosphate (G3P), the diterpenoid dehydroabietinal, pipecolic acid, and the dicarboxylic acid azelaic acid (Wang et al., 2018; Wenig et al., 2019). Systemic effects of *Psa* infection were demonstrated by correlation‐based network analysis as well as independent component analysis. *Psa*‐infection significantly increased the contents of Gln, Arg, pyroglutamic acid, and isocitric acid, but reduced the contents of myo‐inositol, sucrose, glucose, galactose, sorbose, idose, psicose, and mannose in the distant leaves.

## 4 CONCLUSION

This study indicated that *Psa*‐infection resulted in AAs anaplerotic reactions, in turn leading to an extensive C and N reprogramming in various metabolic pathways in leaves, stems, and bleeding saps by inhibiting photosynthesis, C fixation, and TCA cycle (Figure 7). In situ zymography analysis showed that *Psa*‐infection increased *N*‐acetyl‐glucosaminidases (chitinases) activity and was highest at the root tips, indicating that *Psa*‐inoculation accelerated the lignification in kiwifruit root. The concerted metabolic changes between different tissues may be due to a compromise between tissues to response to *Psa*‐infection, which was reflected by pathogen manipulating C/N metabolism and sweet immunity to benefit infection. Host lignin synthesis is a major physical barrier for restricting bacterial infection. To our knowledge, this work is the first integrative analyses of metabolic and biochemical mechanism underlying *Psa* stress in kiwifruit. Psicose, myo‐inositol, and helicon are promising for diagnostic and detailed metabolic analyses.

## 5 MATERIALS AND METHODS

### 5.1 Biological material

Experiments were performed on *A. chinensis* var. *chinensis* cultivar “Hongyang” (*Psa* susceplible) and *A. chinensis* var. *deliciosa* cultivar “Jinkui” (*Psa* resistant) using 3‐year‐old potted plants (75 cm diameter), which were grown in greenhouse of Anhui Agricultural University (31°51′6″, 117°15′26″) at 15 ± 3°C and relative humidity of 85 ± 10%, with long‐day photoperiod (16‐hr light/8‐hr dark) for growth, and short‐day photoperiod (8‐hr light/16‐hr dark) for *Psa* infection.

*P. syringae* pv. *actinidiae* (*Psa*) strain JF8 (CCTCC AB2018305, biovar 3) was used for artificial inoculation, which isolated from cultivar “Jinfeng” in Yuexi, Anhui, P.R China. The inocula were cultured in nutrient‐sucrose agar (NSA) at 25°C for 48 hr, and then were resuspended (1–2 × 10^7^ CFU/ml) in 10 mM MgCl_2_. The strains were inoculated via the stomata into abaxial side of leaflet with a 1 ml sterilized plastic syringe without needle. The 10 mM MgCl_2_ was used as a mock treatment.

Twenty days post‐inoculation (dpi), leaves and stems of “Hongyang” were cut from plants at the base of the lamina, and bleeding saps were collected from cutting branches as described previously (Ferguson, 1980). Those “Hongyang” samples were labeled as A: JY 1–6 (mock leaves), B: JZ 1–6 (mock stems), C: BY 1–6 (local leaves), D: BZ 1–6 (local stems), E: BJY 1–6 (long distances of non‐infected leaves); F: JSL 1–3 (mock shoot bleeding saps); and G: BSL 1–3 (local shoot bleeding saps). Experiments were repeated for six times with biologically independent samples except three for F and G.

### 5.2 NMR analysis

The samples were ground with liquid nitrogen, and then freeze‐dried, extracted by 50% methanol, and sonicated (4 s on/off cycling, for 8 cycles). Samples were centrifuged at 8,500 ***g*** for 15 min. The supernatant was lyophilized, then re‐dissolved in 450 μl water. Subsequently, 50 μl DSS Standard Solution (DSS; Anachro, Canada) was added into 450 μl aqueous layer, and the mixture was homogenized for 10 s. Samples were transferred into 5 mm NMR tube. The metabolites were analyzed by Bruker AV III 600 MHz spectrometer with 600.13 MHz.

The collected free induction decay (FID) signal was automatically zero filled and Fourier transformed in processing module in Chenomx NMR Suite 8.3 (Chenomx Inc.). And then, the data were phased, baseline corrected, and analyzed with DSS as the internal standard in Chenomx Processor against Chenomx Compound Library. NMR analysis was carried out with reference compounds as internal standard. Public databases were used to identify metabolites with chemical shifts that were not available in our database (BMRB: www.bmrb.wisc.edu and MDL: http://www.liu.se/hu/mdl/main/). Six independent biological replicates were performed for each group.

### 5.3 GC‐MS analysis

The metabolic profiling was analyzed according to the method described in previous studies with minor modifications using Agilent 5975C MSD mass spectrometer (Agilent Technologies) coupled with Agilent 7890A GC system containing a fused‐silica capillary column (30 m × 0.25 mm i.d., 0.25 μm DB‐5 MS stationary phase). Samples were extracted and analyzed as described by Moros et al. (2017). For metabolite identification, all components' MS spectra were matched against Golm Metabolome Database (GMD). Six independent biological replicates were performed for each group.

### 5.4 Phenolic compound analysis

One gram fresh weight (FW) of leaf tissue was ground to a fine powder in liquid nitrogen and extracted as described by Gómez‐Romero et al. (2010). Samples were analyzed on an Agilent 1260 (Agilent Technologies) equipped with a diode array detector (DAD). A Nucleosil Agilent Proshell 120 EC‐C_18_ (4.6 × 100 mm, 2.7 μm) was used with the following gradient elution program (solution A, 0.1% formic acid, and solution B, acetonitrile with 0.1% formic acid): 0 min, 5% B; 30 min, 15% B; 60 min, 22% B; and 70 min, 5% B. The flow rate was 0.8 ml/min, and the injection volume was 10 μl. The phenolic compounds were detected at 280 nm. Identification was carried out by comparing reference phenolic compounds, that is, arbutin, gallic acid, chlorogenic acid, caffeic acid, epicatechin, vanillin, *p*‐couaric acid, ferulic acid, 1,2‐dihydroxybenzene, phloridzindihydrate, naringin, trans‐cinnamic acid, and quercetin (Sigma‐Aldrich). Results were presented as mg/g.FW. Three biological replicates were performed for each group.

### 5.5 Antimicrobial activity of phenolic compounds

The 20 μl *Psa* strain JF8 suspension (10^8^ CFU/ml) and different concentrations (40, 80, 160, 320, and 640 μg/ml) of *p*‐coumaric acid, ferulic acid, caffeic acid, or epicatechin were mixed in 4.98 ml KB liquid medium in tube. Bacterial strain was grown for 24 hr at 25°C with 180 rpm. Growth concentration of *Psa* was measured spectrophotometrically at OD_600_ nm. Three biological replicates were performed for each group.

### 5.6 Analysis of PAL and POD activity and lignin content

PAL and POD activities in leaves were quantified at 1, 2, 4, and 8 days post‐infection (dpi). Samples were analyzed using Phenylalanine ammonialyase and Polyphenol Oxidase Kit (Nanjing Jiancheng Bioengineering Institute, China). The lignin content was quantified by the method described by Syros et al. (2004). Three biological repetitions for each group.

### 5.7 In situ zymography, image processing, and analysis

Seeds of *A. chinensis* var. *chinensis* were sterilized in NaClO 2% for 10 min and rinsed four times with sterilized water. The seeds were germinated on agar medium for 20 days, and then seedlings were transplanted in new MSR medium (10 × 10 cm). *P. syringae* pv. *actinidiae* inoculation method as described above. MUF‐cellulose and MUF‐*N*‐acetyl‐glucosaminide were used to measure the distribution and activity of chitinases. The methods of in situ zymography were followed the protocol improved by Razavi et al. (2016) and Liu et al. (2017). The experiment was repeated three times. Three biological repetitions for each group.

### 5.8 Statistical analysis

Supervised models of PLS‐DA were constructed. The PLS‐DA loadings plots along with the VIP were used to ascertain the statistical weight that spectral variables/bins accounted for.

All the data were assayed through a Student's *t* test and significant differences were reported.

## CONFLICT OF INTEREST

The authors declare no conflicts of interest.

## AUTHOR CONTRIBUTIONS

P.L. and Y.Z. conceived and designed the research; Y.L. performed the experiments together with X.W provided technical assistance on the experiment; P.L., X.W., and Y.L. analyzed the data and wrote the article.

## Supporting information

Fig S1‐S4

Table S1

Table S2

Table S3

Supplementary Material
