# Supplementary material for: Metabolic profiling reveals local and systemic responses of kiwifruit to Pseudomonas syringae pv. actinidiae
Source: Plant Direct. 2020 Dec 16;4(12):e00297. doi: 10.1002/pld3.297 (PMC7739878; doi:10.1002/pld3.297)
Supplement: Supplementary file 1 — Fig S1‐S4 [file PLD3-4-e00297-s001.docx]

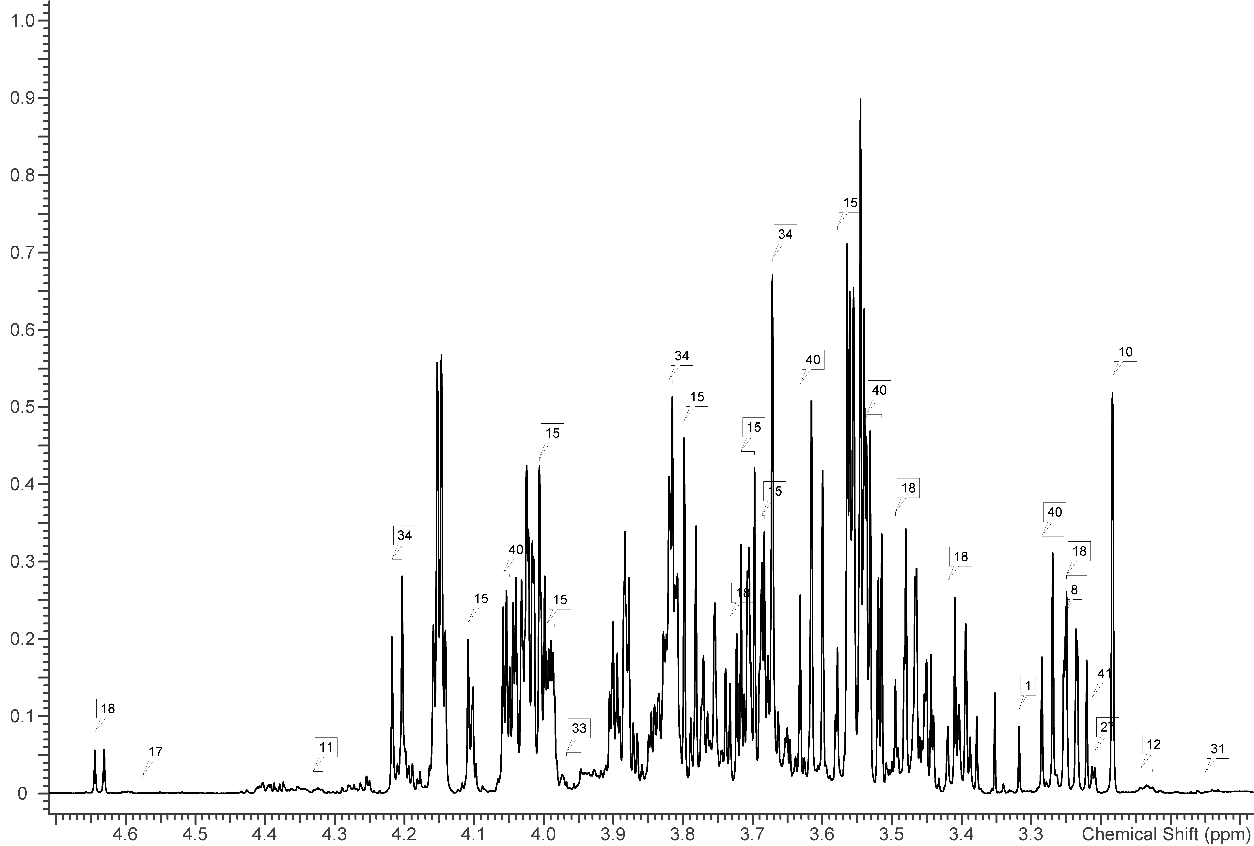


**Supplemental Figure S1. A representative ^1^H NMR spectrum of kiwifruit instems.** (1) 1,3-dimethylurate, (2) 2-hydroxybutyrate, (3) acetate, (4) alanine (Ala), (5) arginine (Arg), (6) asparagine (Asn),(7) aspartate (Asp), (8) betaine, (9) chlorogenate, (10) choline, (11) epicatechin, (12) ethanolamine, (13) ferulate, (14) formate, (15) fructose, (16) fumarate, (17) galactose, (18) glucose, (19) glutamate (Glu), (20) glutamine (Gln), (21) isobutyrate, (22) isoleucine (Ile), (23) lactate, (24) leucine (Leu), (25) lysine (Lys), (26) methanol, (27) o-phosphocholine, (28) phenylalanine (Phe), (29) pipecolate, (30) proline (Pro), (31) putrescine, (32) pyruvate, (33) serine (Ser), (34) sucrose, (35) threonine (Thr), (36) trehalose, (37) UDP-galactose, (38) UDP-glucose, (39) valine (Val), (40) myo-inositol, (41) sn-glycero-3-phosphocholine


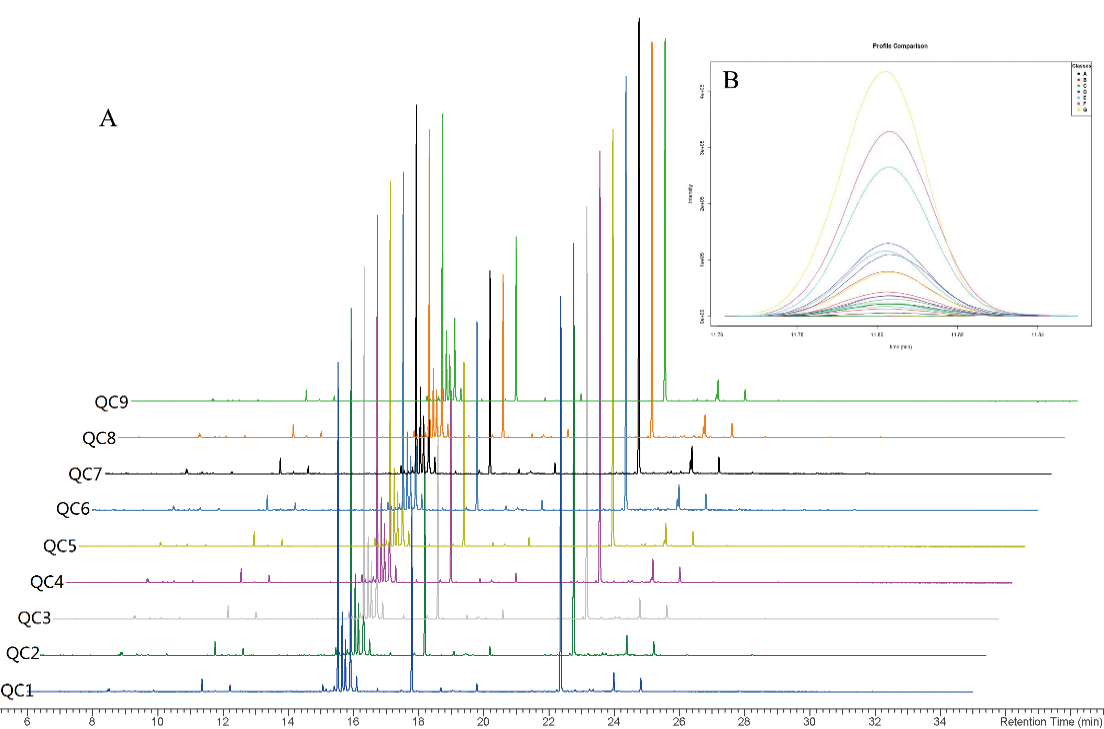
**Supplemental Figure S2. GC-MS total ion chromatogram (A) and Agilent ChromStation retention time alignment (B) of a kiwifruit leaves extract.**


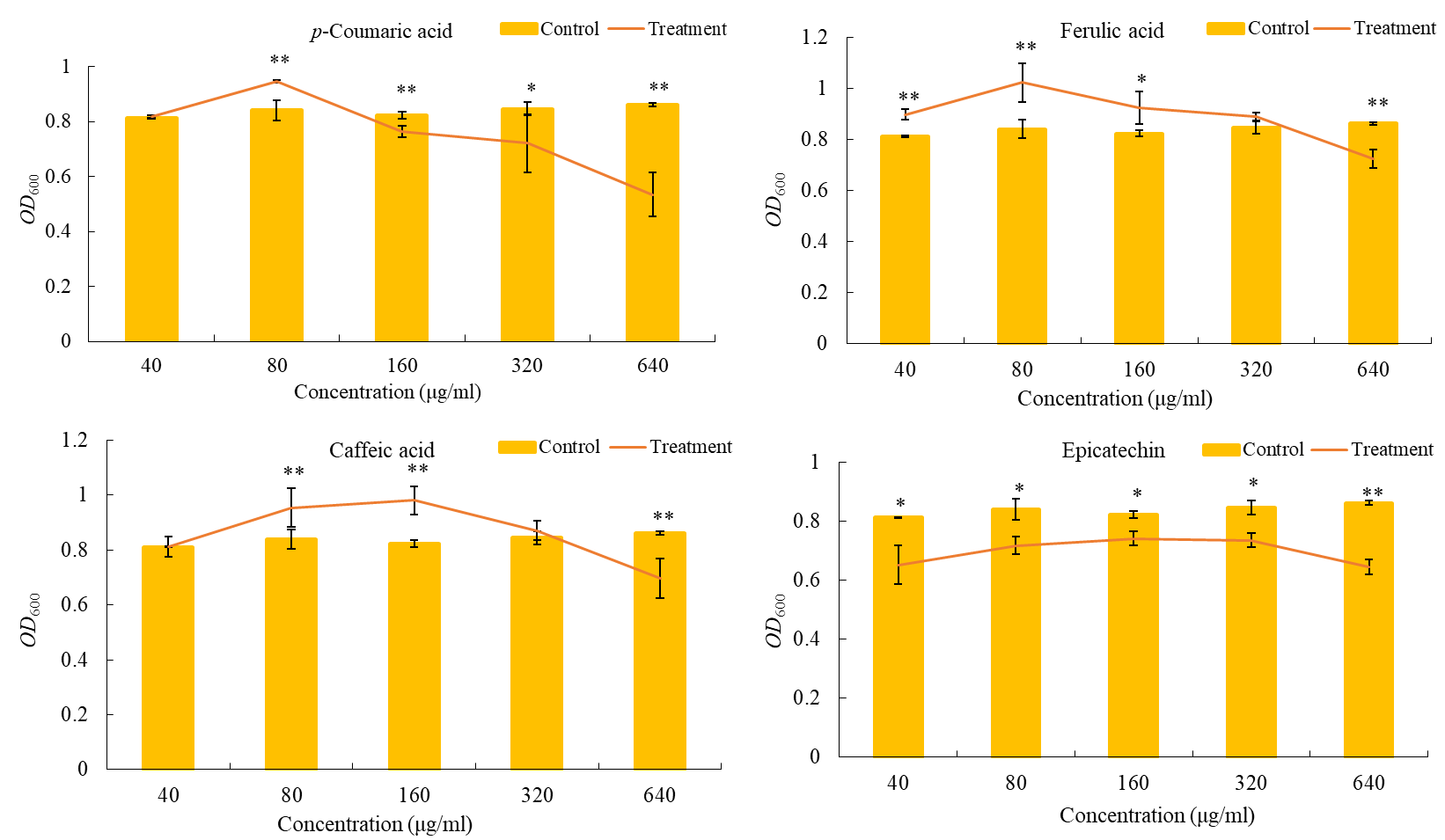


**Supplemental Figure S3. Effects of the phenolic compounds of p-coumaric acid,** **ferulic acid,** **caffeic acid and** **epicatechin on growth of Psa bacterium**


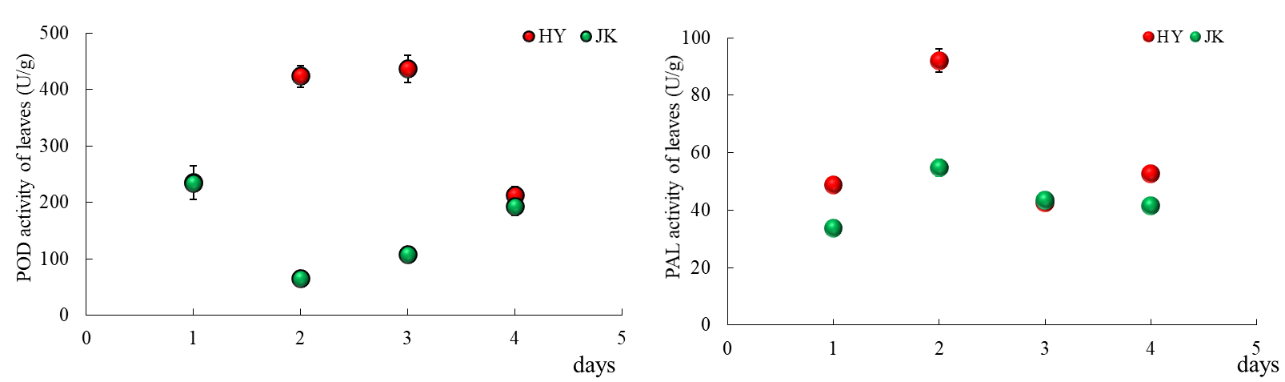


**Supplemental Figure S4. Enzyme activity of PAL and POD response to Psa bacterium in ‘Hongyang (HY)’ and ‘Jinkui (JK)’**
