# Supplementary material for: Metabolic profiling reveals local and systemic responses of kiwifruit to Pseudomonas syringae pv. actinidiae
Source: Plant Direct. 2020 Dec 16;4(12):e00297. doi: 10.1002/pld3.297 (PMC7739878; doi:10.1002/pld3.297)
Supplement: Supplementary file 5 — Supplementary Material [file PLD3-4-e00297-s005.pdf]

**Dear editor,**

**We appreciate the constructive suggestions about our manuscript. The following letter is a detailed revised version of the manuscript, we have adopted most of the points reviewers have mentioned in our revised manuscript. The changes made to the manuscript have been highlighted in red. Please contact us if you had any further questions.**

•Reviewer #2:

The paper has improved substantially. However, some aspects still need clarification.
**REPLY: Authors thank the reviewer for the positive remarks, constructive suggestions and comments from the previous review.**
It is absolutely required that all the statements mentioned in the paper are confirmed on solid data. Therefore, avoid statements like this:" Moreover, our present transcriptomic data suggests that Psa inhibits photosynthesis in 'Hongyang' (data not shown)." You can mention something like " Recent transcriptomic data..."
**REPLY: Corrections were made in the revised manuscript.**

Additionally, please check the comments of the previous reviewer in what concerns the lack of certain comparisons between susceptible and resistant cultivars. Again, the authors must make sure that all the statements are based on solid results or otherwise are just assumptions.
**REPLY: In this study, we combined $^{1}$H-NMR spectroscopy with GC-MS to reveal local and systemic responses of kiwifruit to *Pseudomonas syringae* pv. *actinidiae*. Limited by funds and plant material, we only used 'Hongyang' as the samples. Recently, we realized this problem and planned to verified the above data by transcriptome with susceptible and resistant cultivars.**

Additionally, please check the format of your PDF. We cannot accept it in the current form.
**REPLY: The authors are sorry for the inconvenience (because of merged the Supplemental Table S1-3). Corrections were made in the revised manuscript.**
